# Human granulocyte-colony stimulating factor (G-CSF)/stem cell factor (SCF) fusion proteins: design, characterization and activity

**DOI:** 10.7717/peerj.9788

**Published:** 2020-08-21

**Authors:** Gitana Mickiene, Indrė Dalgėdienė, Gintautas Zvirblis, Zilvinas Dapkunas, Ieva Plikusiene, Ernesta Buzavaite-Verteliene, Zigmas Balevičius, Audronė Rukšėnaitė, Milda Pleckaityte

**Affiliations:** 1 Institute of Biotechnology, Vilnius University, Vilnius, Lithuania; 2 Profarma UAB, Vilnius, Lithuania; 3 Department of Physical Chemistry, Faculty of Chemistry and Geosciences, Vilnius University, Vilnius, Lithuania; 4 Plasmonics and Nanophotonics Laboratory, Department of Laser Technology, Center for Physical Sciences and Technology, Vilnius, Lithuania

**Keywords:** Fusion proteins, G-CSF, SCF, Linker, Hematopoiesis, Cytokines, Absolute neutrophil count, Protein purification, Activity

## Abstract

**Background:**

Stem cell factor (SCF) and granulocyte-colony stimulating factor (G-CSF) are well-characterized vital hematopoietic growth factors that regulate hematopoiesis. G-CSF and SCF synergistically exhibit a stimulatory effect on hematopoietic progenitors. The combination of G-CSF and SCF has been used for mobilization of peripheral blood progenitor cells in cancer and non-cancerous conditions. To overcome challenges connected with the administration of two cytokines, we developed two fusion proteins composed of human SCF and human G-CSF interspaced by an alpha-helix-forming peptide linker.

**Methods:**

The recombinant proteins SCF-Lα-GCSF and GCSF-Lα-SCF were purified in three steps using an ion-exchange and mixed-mode chromatography. The purity and quantity of the proteins after each stage of purification was assessed using RP-HPLC, SDS-PAGE, and the Bradford assays. Purified proteins were identified using high-performance liquid chromatography/electrospray ionization mass spectrometry (HPLC/ESI-MS) and the Western blot analyses. The molecular weight was determined by size exclusion HPLC (SE-HPLC). The activity of heterodimers was assessed using cell proliferation assays in vitro. The capacity of recombinant fusion proteins to stimulate the increase of the absolute neutrophil count in rats was determined in vivo. The binding kinetics of the proteins to immobilized G-CSF and SCF receptors was measured using total internal reflection ellipsometry and evaluated by a standard Langmuir kinetics model.

**Results:**

The novel SCF-Lα-GCSF and GCSF-Lα-SCF proteins were synthesized in *Escherichia coli*. The purity of the heterodimers reached >90% as determined by RP-HPLC. The identity of the proteins was confirmed using the Western blot and HPLC/ESI-MS assays. An array of multimeric forms, non-covalently associated dimers or trimers were detected in the protein preparations by SE-HPLC. Each protein induced a dose-dependent proliferative response on the cell lines. At equimolar concentration, the heterodimers retain 70–140% of the SCF monomer activity (*p* ≤ 0.01) in promoting the M-07e cells proliferation. The G-CSF moiety in GCSF-Lα-SCF retained 15% (*p* ≤ 0.0001) and in SCF-Lα-GCSF retained 34% (*p* ≤ 0.01) of the monomeric G-CSF activity in stimulating the growth of G-NFS-60 cells. The obtained results were in good agreement with the binding data of each moiety in the fusion proteins to their respective receptors. The increase in the absolute neutrophil count in rats caused by the SCF-Lα-GCSF protein corresponded to the increase induced by a mixture of SCF and G-CSF.

## Introduction

Hematopoiesis is a complex process controlled by many cytokines and growth factors (Mehta, Malandra & Corey, 2015; Hoggatt, Kfoury & Scadden, 2016). Granulocyte-colony stimulating factor (G-CSF) and stem cell factor (SCF) are among the key members of the hematopoietic growth factor family (Morstyn et al., 1994; Hassan & Zander, 1996; Broudy, 1997; Mehta, Malandra & Corey, 2015). Defects in G-CSF have deleterious effects on the organisms. Mice lacking G-CSF had chronic neutropenia with neutrophil levels reaching 20–35% of the age-matched wild type controls (Lieschke et al., 1994). G-CSF affects the bone marrow that stimulates the production of neutrophilic granulocytes and their release into the bloodstream (Arai et al., 2001). The G-CSF receptor (GCSF-R), which belongs to the class I cytokine receptors, does not possess an intrinsic tyrosine kinase activity (Demetri & Griffin, 1991). Binding of G-CSF to its receptor activates various signaling cascades via protein phosphorylation (Fujii, 2007). GCSF-R performs signaling functions as a homodimer (Horan et al., 1996; Layton & Hall, 2006). The G-CSF molecule and GCSF-R interacts with 2:2 stoichiometry (Tamada et al., 2006).

Mice with mutations in SCF and its receptor c-kit have a deficiency in blood neutrophils, germ, and tissue mast cells (Ruscetti et al., 1976; Flanagan, Chan & Leder, 1991; Brannan et al., 1991). The complete absence of SCF leads to embryonic anemia and lethality, thereby identifying SCF as a critical regulator of erythropoiesis (Huang et al., 1990; Khodadi et al., 2016). SCF in combination with other cytokines acts on hematopoietic cells and displays synergistic responses that induce expansion and development of hematopoietic lineages (McNiece, Langley & Zsebo, 1991; Lennartsson, Shivakrupa & Linnekin, 2004; Antonchuk et al., 2004; Raju et al., 2003). SCF is a ligand of the receptor c-kit, which has an intrinsic tyrosine kinase activity (Reber, Da Silva & Frossard, 2006). SCF binding to c-kit causes autophosphorylation of the receptor that leads to activation of signaling cascades including the JAK/STAT and Src kinase pathways (Huang et al., 1990; Ho et al., 2017). SCF is found soluble in blood serum and displayed on the cell surface (Flanagan, Chan & Leder, 1991). Soluble SCF exists as a monomer and homodimer (Lu et al., 1995), but the activity of the covalent dimer is found to be higher than that of monomeric SCF (Nocka et al., 1997).

Granulocyte-colony stimulating factor has been intensively used for treatment of cancer therapy-induced neutropenia (Morstyn et al., 1988; Sheridan et al., 1992; Paul et al., 2014) and other neutropenic conditions (Becker-Cohen et al., 2015; Mehta, Malandra & Corey, 2015; Skokowa et al., 2017). It is marketed as an *Escherichia coli* derived recombinant protein (filgrastim), including glycosylated (lenograstim) and PEGylated (pegfilgrastim) biobetters (Bönig et al., 2001; Molineux, 2004). G-CSF in combination with SCF showed a sustained increase of peripheral blood progenitor cells for autologous transplantation (Moskowitz et al., 1997; Shapiro et al., 1997). Recombinant SCF, marketed as Ancestim, in combination with G-CSF allowed successful collection of CD34^+^ progenitors in the peripheral blood of poorly mobilizing cancer patients (Lapierre et al., 2011). Ancestim is not administered alone (Stemgen® (ancestim), Product Monograph, 2009).

The synergistic activity of cytokines promoted the development of fusion proteins (Schuh & Morrissey, 1999; Lee et al., 2003; Chen et al., 2005; Ng & Galipeau, 2015). Fusion cytokines are attractive for therapeutic applications because their concerted activity enhances the effect of the separate moieties and even confer novel functions. The administration of a fusion protein reduces the adverse effects of repeated injections of cytokines.

In this study, we described the development of two heterodimeric fusion proteins composed of human SCF and human G-CSF connected via a peptide linker, which ensured the activity of two monomers in the heterodimeric molecule (Arai et al., 2001; Mickiene et al., 2017; Balevicius et al., 2019). The heterodimers were purified, characterized, and compared with monomeric human G-CSF and SCF. The fusion proteins possessed the receptor binding activity that resulted in cell proliferation. The SCF-Lα-GCSF protein exhibited increased biological activity in vivo comparable to that of SCF plus G-CSF.

## Materials and Methods

### Generation of recombinant fusion proteins

**Figure 1:**
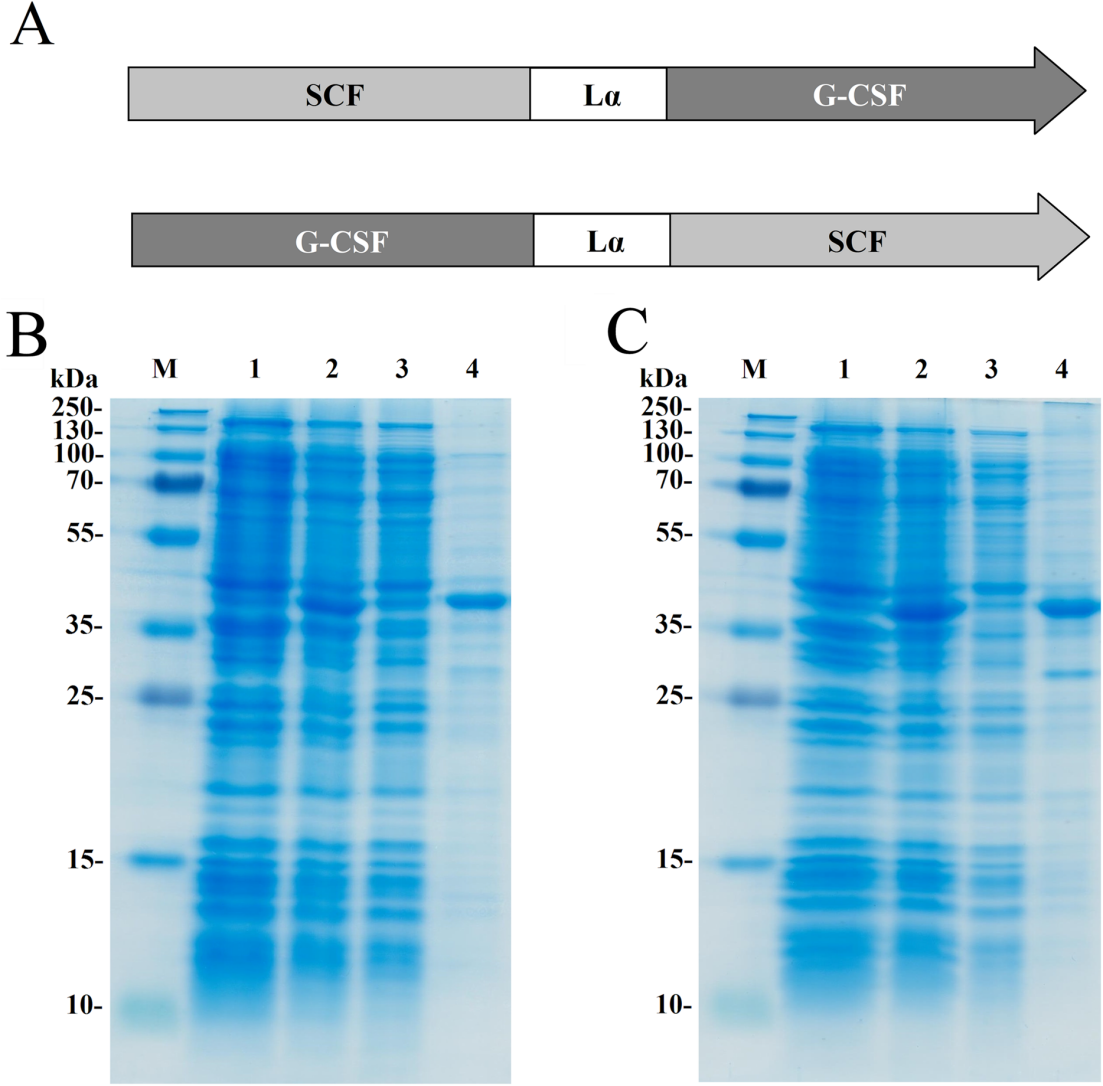
Expression of the fusion proteins in *E. coli*. (A) Schematic representation of the SCF-La-GCSF and GCSF-La-SCF constructs. The sequence of the Lα linker is presented in the Materials and Methods section. SDS-PAGE of the recombinant SCF-Lα-GCSF (B) and GCSF-Lα-SCF (C) proteins in *E. coli* cell lysates. Lane 1, cell lysates before induction; lane 2, cell lysates after induction; lane 3, soluble fraction of cell lysates; lane 4, insoluble fraction of cell lysates; lane M, prestained molecular weight marker (Thermo Fisher Scientific, Waltham, MA, USA).

Two recombinant proteins SCF-Lα-GCSF and GCSF-Lα-SCF were generated by a covalent fusion of SCF and G-CSF molecules. The DNA fragment coding for a human SCF and Lα linker was synthesized at Integrated DNA Technologies (USA). All primers were obtained from Metabion (Germany). The *scf* gene having NdeI at the 5′-end and Kpn2I/BamHI sites at the 3′-end was amplified using 5′-GTGCATATGGAAGGTATCTGTCGT and 5′-GGATCCAAGTCCGGAAGCAGCAACCGGCGGCAGC primers. All amplified sequences were verified by sequencing. The DNA fragment was fused with the Kpn2I/BamHI digested DNA fragment coding for the Lα linker sequence SGLEA(EAAAK)_4_ALEA(EAAAK)_4_ALEGS (Arai et al., 2001). The synthetic human *gcsf* gene (Integrated DNA Technologies, Coralville, IA, USA) having the BamHI and HindIII sites at the 5′- and 3′-ends, respectively, was fused to the 3′-end of the linker Lα sequence. The construct obtained by a fusion of the *scf* and *g-csf* genes interspaced by the linker Lα sequence was named SCF-Lα-GCSF (Fig. 1A).

The *scf* gene was amplified to introduce the BamHI and HindIII sites at the 5′-and 3′-ends of the PCR fragment using primers 5′-TGGATCCGAAGGGATCTGCCGTAATCG and 5′-TAAGCTTAGGCTGCAACAGGGGG, respectively. The copy of the human *g-scf* gene was cut out with the enzymes BamHI and HindIII from the plasmid bearing two copies of the *g-csf* gene interspaced by the Lα sequence (Mickiene et al., 2017). The BamHI/HindIII digested DNA fragment coding for SCF was fused with the DNA construct bearing the *g-scf* gene and the Lα linker sequence. The resulting construct was named GCSF-Lα-SCF (Fig. 1A).

The DNA fragments coding for SCF-Lα-GCSF and GCSF-Lα-SCF were cloned into the expression vector pET21b(+) (Merck Millipore, Darmstadt, Germany). The resulting plasmids pET21-SCF-GCSF and pET21-GCSF-SCF were transformed into *E. coli* BL21(DE3) and BL21(DE3)STAR strains (both strains obtained from Merck Millipore, Darmstadt, Germany), respectively.

### Expression of recombinant fusion proteins in *E. coli*

The cells were grown overnight in 25 mL of the LB broth supplemented with 100 µg/mL ampicillin. An overnight culture was diluted (ratio 1:100) in 400 mL minimal salts (M9) medium supplemented with 0.5% yeast extract, 0.4% glucose, 2 mM MgSO_4_ and 100 µg/mL ampicillin, and cultivated at 37 °C with agitation to OD_600_ of 0.8. The recombinant protein expression was induced by 1 mM isopropyl-β-D-thiogalactoside (IPTG) for 3 h at 37 °C. The cells were collected by centrifugation at 4,000×*g* for 30 min at 4 °C. The harvested biomass was disrupted by sonication. The supernatant (soluble fraction) and the cell pellet (insoluble fraction) were analyzed by polyacrylamide gel electrophoresis (SDS-PAGE) under reducing conditions.

### Purification of recombinant fusion proteins

The isolation and purification of inclusion bodies (IB) from the harvested biomass (12 g wet weight) was performed as previously described (Zaveckas et al., 2003). Briefly, the cell pellets were solubilized in 120 mL of a buffer containing 50 mM Tris-HCl (pH 8.0), 0.5 mM 1,4-dithiothreitol (DTT) and 8 M urea. The solution was stirred at 4 °C for 2 h and centrifuged at 30,000×*g* for 25 min. Refolding of the recombinant proteins was initiated by rapid dilution of the denatured/reduced proteins into the 50 mM Tris-HCl (pH 8.0) buffer supplemented with oxidized glutathione (GSSG) until the concentration of 2 M urea was reached. The final molar ratio of GSSG to DTT was kept 1:5 in the mixture. The renaturation reaction was carried out for 24 h at 4 °C with gentle stirring. The solution was then centrifuged for 25 min at 30,000×*g*.

A three-step purification scheme for recombinant proteins was performed on the ÄKTA pure 150 system (GE Healhcare, Uppsala, Sweden). The refolded soluble protein was loaded on a DEAE Sepharose FF column (GE Healthcare, Uppsala, Sweden) equilibrated with 50 mM Tris-HCl (pH 7.5). The proteins were eluted by a step-wise elution steadily increasing the concentration of NaCl from 0 to 0.5 M. The collected fractions containing recombinant proteins were subsequently loaded onto a CHT ceramic hydroxyapatite, Type II column (Bio-Rad Laboratories, Hercules, CA, USA), equilibrated with a 50 mM Tris-HCl buffer (pH 7.2). The column was washed with 5 mM NaH_2_PO_4_ (pH 7.2) containing 0.1 M NaCl, and the proteins were eluted using a gradient of NaH_2_PO_4_ (5–500 mM). The target protein-containing fractions were pooled and diluted with 20 mM sodium acetate to pH 4.7 and then loaded on an SP Sepharose FF column (GE Healthcare, Uppsala, Sweden) equilibrated with 20 mM sodium acetate (pH 4.7). The column was washed with the equilibration buffer and the proteins were eluted with the NaCl gradient using the equilibration buffer supplied with 500 mM NaCl.

Fractions containing the highest amount of pure protein were pooled and transferred to the storage buffer composed of 20 mM acetic acid/NaOH (pH 4.0) by diafiltration using 10-kDa centrifugal filter devices (AmiconUltra-15; Millipore, Burlington, MA, USA). The protein solution was filtered through 0.2 μm Acrodisc Units with Mustang E membrane (Pall Corporation, New York, NY, USA) for endotoxin removal. The purified protein solutions were stored at +4 °C.

### RP-HPLC analysis

The purity of the recombinant heterodimeric proteins after each stage of purification was assessed by both RP-HPLC and SDS-PAGE analysis. RP-HPLC was carried out using an Alliance e2695 HPLC system (Waters, Milford, MA, USA) with UV absorbance detection at 215 nm. The SCF-Lα-GCSF and GCSF-Lα-SCF proteins from IBs extracts, their folding intermediates, and fractions derived from the chromatography columns were analyzed on a C_18_ reverse-phase column (Zorbax 300SB-C18, 4.6 × 250 mm; Agilent Technologies, Santa Clara, CA, USA). The chromatographic separation of the proteins was performed in acetonitrile gradient (mobile phase A—0.1% trifluoroacetic acid (TFA) in water, mobile phase B—9.9% water, 90% acetonitrile, and 0.1% TFA) at a flow rate 1 mL/min, as follows: (1) initial equilibration at 10% B, (2) a 5-min gradient to 58% B, (3) a 74-min gradient to 81% B, (4) a 3-min gradient to 90% B, (5) a 4-min isocratic elution at 90% B, (6) a 3-min gradient to 10% B, and a final (7) 5-min isocratic elution at 10% B. The temperature of the column was maintained at 30 °C. SDS-PAGE was performed on a slab gel containing 15% polyacrylamide by the method of Laemmli (1970).

### Western blotting

The purified heterodimeric proteins SCF-Lα-GCSF and GCSF-Lα-SCF, and monomeric proteins G-CSF (filgrastim, Sicor Biotech, Teva, Petah Tikva, Israel) and SCF (#AB179506; Abcam, Cambridge, UK) resolved by SDS-PAGE were transferred onto the Immobilon-P PVDF membranes (Merck Millipore, Tullagreen, Carrigtwohill, Cork, Ireland). The membranes were blocked with 5% BLOT-QuickBlocker (Calbiochem, San Diego, CA, USA) in PBS supplemented with 0.1% Tween 20 (PBS-T) for 2 h. The blocking solution was removed and the membranes were incubated for 1 h either with 1:2,000 PBS-T diluted monoclonal antibody (clone no. 5D7) against human G-CSF (Abcam, Cambridge, UK) or 1:1,000 diluted polyclonal antibody against human SCF (Invitrogen, Carlsbad, CA, USA and Thermo Fisher Scientific, Waltham, MA, USA). The membranes were washed with PBS-T and then incubated for 1 h either with 1:4,000 diluted rabbit anti-mouse IgG conjugated to horseradish peroxidase (Invitrogen, Carlsbad, CA, USA and Thermo Fisher Scientific, Waltham, MA, USA) or 1:1,000 diluted goat anti-rabbit IgG conjugated to horseradish peroxidase (Invitrogen, Carlsbad, CA, USA and Thermo Fisher Scientific, Waltham, MA, USA). The enzymatic reaction was developed using a tetramethylbenzidine chromogenic substrate (Sigma–Aldrich, St. Louis, MO, USA).

### Size-exclusion HPLC

The purified fusion proteins and monomeric G-CSF and SCF were injected into a TSK-gel G3000 SWXL column (7.8 × 300 mm, 5 µm, Tosoh Bioscience, Tokyo, Japan) connected to an Alliance e2695 HPLC system. The proteins were eluted with an isocratic mobile phase of 0.1 M Na_2_HPO_4_, 0.1 M Na_2_SO_4_ (pH 7.2) and a flow rate 0.6 mL/min (22 °C). The molecular weight of proteins was estimated based on the retention time of the Protein standard mix for SEC (Sigma–Aldrich, St. Louis, MO, USA).

### Molecular mass determination

The molecular mass of SCF-Lα-GCSF and GCSF-Lα-SCF was determined by the integrated method of high-performance liquid chromatography/electrospray ionization mass spectrometry (HPLC/ESI-MS). The protein samples were diluted with an aqueous solution containing 1% formic acid (FA) and 2% acetonitrile to the concentration of 0.1 µg/µL. The samples of 10 μL were loaded onto a C_8_ reverse-phase column (Poroshell 300SB-C8, 2.1 × 75 mm; Agilent Technologies, Santa Clara, CA, USA). Chromatographic separation of proteins was performed in an acetonitrile gradient (mobile phase A—1% FA in water, mobile phase B—1% FA in acetonitrile) at a flow rate 0.4 mL/min on an Agilent 1290 Infinity HPLC system coupled to an Agilent Q-TOF 6520 mass spectrometer (Agilent Technologies, Santa Clara, CA, USA), as follows: (1) initial equilibration at 2% B, (2) a 5-min gradient to 98% B, (3) a 1-min isocratic elution at 98% B, (4) a 2 min gradient to 2% B, and a final (5) a 1-min isocratic elution at 2% B. The temperature of the column was maintained at 30 °C. The mass analyzer was set to 100–3,200 m/z range in a positive ionization mode. The data were analyzed with Agilent MassHunter Workstation Software.

### Endotoxin quantification

Endotoxin contamination of the recombinant protein preparations was detected using the Pyrotell Gel-Clot endotoxin testing kit (Cape Cod, MA, USA) according to the manufacturer’s instructions. The sensitivity of the Limulus amebocyte lysate-based assay was 0.125 EU/mL. The determined endotoxin level in the purified proteins was ≤0.25 EU/mL or ≤0.29 EU/mg.

### Total internal reflection ellipsometry measurements

The binding kinetics assay of the purified heterodimers and monomeric SCF to a surface-immobilized G-CSF and SCF receptors was performed by total internal reflection ellipsometry (TIRE). The binding assays of monomeric G-CSF and SCF-Lα-GCSF to GCSF-R were described in Balevicius et al. (2019). GCSF-R and c-kit used for TIRE are chimeric proteins (both obtained from Abcam). The extracellular domain of the receptors was fused to the Fc region of human immunoglobulin (IgG1).

The experimental setup consisted of a spectral ellipsometer M-2000X, J. A. Woollam (Lincoln, Dearborn, MI, USA) with a rotating compensator operating in a spectral range of 200–1,000 nm. Refractive index matching fluid was used to obtain optical contact between the BK7 70 glass prism and 1 mm thick BK7 glass slide covered by a layer of chromium and gold (BK7-glass/Cr-Au, XanTecbioanalytics GmbH, Duesseldorf, Germany) for surface plasmon excitation. The reaction chamber with a volume of 0.028 mL was positioned underneath the cell layer of the slide. Two gold sensor disks for immobilization of GCSF-R and c-kit were prepared as described previously (Balevicius et al., 2014, 2019) with slight modifications. Briefly, the BK7-glass/Cr-Au slides were cleaned using the BAL-TEC SCD 050 Sputter Coater plasma cleaner, washed with methanol for 10 min, and then rinsed with hexane for 5 min. The self-assembled monolayer (SAM) was prepared by immersing the surface plasmon resonance (SPR) chips into a 1 mM 11-mercaptoundecanoic acid (11-MUA) solution in methanol for 18 h followed by a rinse with methanol. Further modification of the glass slide, including covalent immobilization of protein G, were performed as described previously (Balevicius et al., 2019). The glass slide coated with the SAM-immobilized protein G via the Fc region was incubated in PBS (pH 7.4), containing 0.0532 μM of GCSF-R or 0.0602 μM of c-kit for 60 min. The slides BK7-glass/Cr-Au/MUA/Protein-G/GCSF-R or BK7-glass/Cr-Au/MUA/Protein-G/c-kit were washed with PBS for 10 min to remove an unbound receptor. The TIRE cell was filled with the PBS buffer (pH 7.4) containing 10 µg/mL of each protein. After 30 min incubation, the cell was flushed with PBS (pH 7.4) to remove the non-specifically attached protein layer.

The protein-receptor interactions were analyzed using the CompleteEase software (J. A. Woollam Co., Inc. data, Lincoln, NE, USA). The binding kinetics of heterodimers and monomeric SCF were evaluated by a standard fully reversible Langmuir kinetics model described previously (Balevicius et al., 2019).

### In vitro biological activity

The in vitro biological activity of SCF-Lα-GCSF and GCSF-Lα-SCF was determined using the SCF-dependent human M-07e cell line (Drexler, Zaborski & Quentmeier, 1997) and G-CSF-dependent mouse G-NFS-60 cell line (Weinstein et al., 1986; Matsuda, Shirafuji & Asano, 1989) as described previously (Mickiene et al., 2017). The SCF and G-CSF monomers were used as the reference standards. Briefly, before the assays, the M-07e and G-NFS-60 cells were centrifuged and resuspended at the concentration of 5.0 × 10^7^ cells/mL in the test medium (RPMI 1640 supplemented with a 10% fetal bovine serum, antibiotic gentamicin sulfate and 0.05 mM 2-mercaptoethanol). A total of 50-µL of the test medium was aliquoted into each well of a 96-well tissue culture plate. The purified heterodimers and standard proteins were serially diluted in the test medium. A volume of 50 µL of the diluted protein was added to the wells to the concentrations of 0.004–7.8 pg/mL in technical triplicate. Each protein was tested in at least three independent assays. After the incubation at 37 °C and 5% CO_2_ for 48 h, 20 µL of a tetrazolium salt solution (MTS, 5 g/L) (Promega, Madison, WI, USA) was added to each well of the plate, and the incubation was continued for 3 h under the same conditions. The absorbance of formazan derived from the MTS cleavage by cellular mitochondrial dehydrogenases was measured using a multi-well scanning spectrofluorometer (FluoroMax-4; Horiba Scientific, Piscataway, NJ, USA) at 490 nm. The biological activity of each protein was calculated from the proliferation curves using OriginLab Origin and Microsoft Excel. The proliferation curves were constructed by plotting the log2 dilution of the proteins or standards against the absorbance value at 490 nm. The specific biological activity of the heterodimers was determined using the equations as described previously (Mickiene et al., 2017).

### Biological activity of SCF-Lα-GCSF in vivo

Healthy female Wistar rats (4 months old, weight 250–300 g) were used to assess the biological activity of SCF-Lα-GCSF in vivo. The study with the laboratory animals was approved by the State Food and Veterinary Service of the Republic of Lithuania (approval no. 0182). The rats were randomized to different test groups with 3–5 rats in each group. Five groups of rats received a single subcutaneous injection of the protein preparations, as follows: 500 µg/kg of G-CSF monomer (group 1), 500 µg/kg of SCF monomer (group 2), 500 µg/kg of purified SCF-Lα-GCSF (group 3), 1,000 µg/kg of purified SCF-Lα-GCSF (group 4) and a combination of 500 µg/kg SCF and 500 µg/kg G-CSF (group 5). Group 6 received the sodium acetate buffer. The biological activity of G-CSF in vivo was assessed measuring an absolute neutrophil count (ANC) in the rats of each group. Blood samples were collected from the tail veins at 0, 24, 48 and 72 h after injection. ANC was determined using a microcell counter (hematology analyzer Exigo EOS, Boule Medical AB, Spånga, Sweden).

### Statistical analysis

The data were evaluated using GraphPad Prism software (version 8.0, GraphPad, San Diego, CA, USA). The data were subjected to analysis using the unpaired *t*-test, the one-sample *t*-test, the one-way analysis of variance (ANOVA) combined with the Tukey’s multiple comparison test, and the Shapiro–Wilk normality test for the data that follow a normal distribution. Results are presented as mean ± standard error of mean (SEM) or mean ± standard deviation (SD) of at least 3 independent experiments.

## Results

### Preparation of recombinant heterodimeric proteins SCF-Lα-GCSF and GCSF-Lα-SCF

The fusion protein SCF-Lα-GCSF composed of human SCF and human G-CSF interspaced by a 54-amino-acid flexible linker (Lα) was generated (Fig. 1A). Human G-CSF joined via Lα to the N-termini of human SCF resulted in the GCSF-Lα-SCF protein. The heterodimeric proteins were expressed in *E. coli* cells. The SDS-PAGE analysis showed that both proteins (mol. weight of 42 kDa) were found in the insoluble fraction of the total cell lysate (Figs. 1B and 1C).

**Figure 2:**
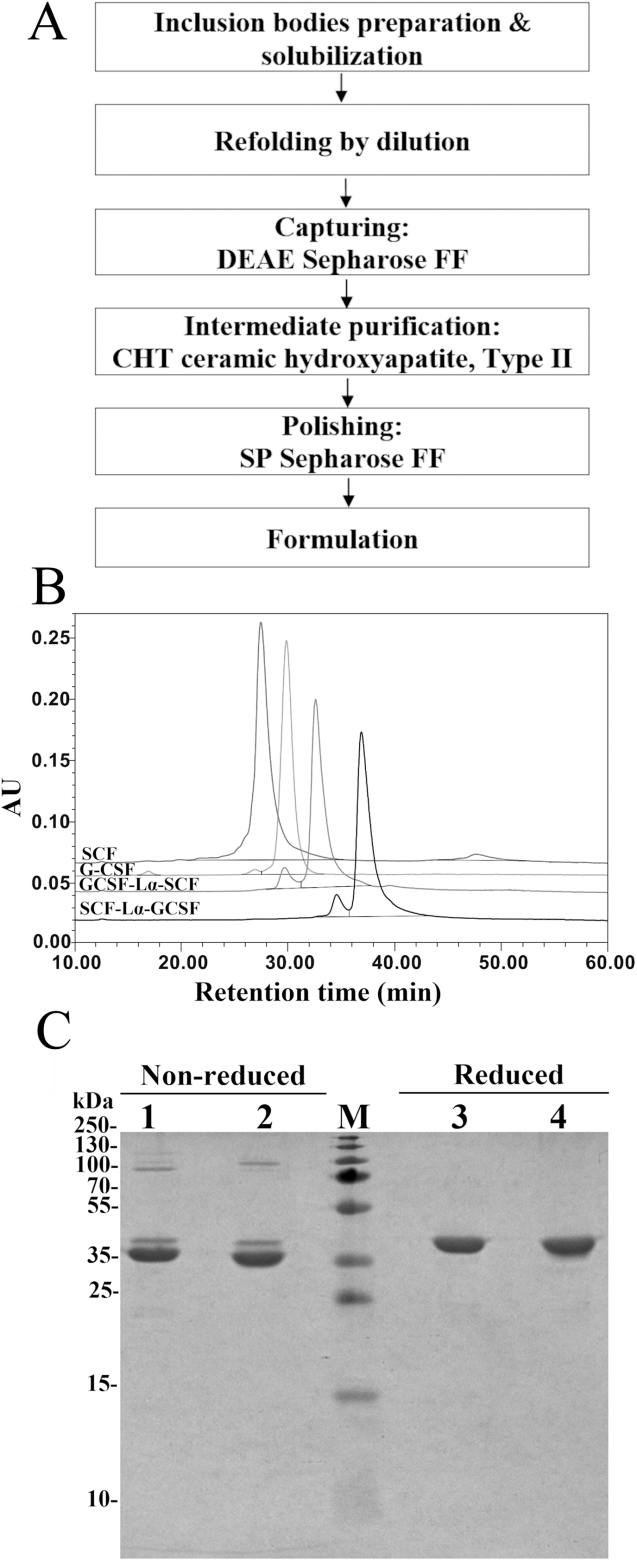
Purification characteristics of the fusion proteins. (A) A flowchart of the purification steps of SCF-Lα-GCSF and GCSF-Lα-SCF. (B) RP-HPLC of the purified fusion proteins. The SCF and G-CSF monomers were used as controls. Fifteen-µg of each protein was loaded onto a Zorbax 300SB-C18 column. Absorbance at 215 nm is reported as AU. (C) SDS-PAGE of the purified fusion proteins under non-reducing and reducing conditions. Lanes 1, 4, SCF-Lα-GCSF; lanes 2, 5, GCSF-Lα**-**SCF; lane M, prestained molecular weight marker (Thermo Fisher Scientific, Waltham, MA, USA).

The RP-HPLC analysis was the main tool throughout the purification to monitor the transition of the reduced protein form into oxidized state. After the solubilization of inclusion bodies, the complete reduction of the disulfide bonds in both proteins was achieved by the addition of 0.5 mM DTT (Fig. S1A). The oxidative refolding of SCF-Lα-GCSF and GCSF-Lα-SCF carried out for 24 h resulted in protein purity of 24.2% and 35.8%, respectively (Table 1; Fig. S1B). The extensive purification of refolded SCF-Lα-GCSF and GCSF-Lα-SCF proteins were achieved using the DEAE Sepharose FF chromatography column. As a result, most of the nonspecific proteins including the aggregates were removed (Figs. S2A–S2D, lane 3) and the purity of the target proteins reached more than 58% as determined by HPLC (Table 1). To reduce other impurities, there were tested different types of chromatographic media including CM Sepharose FF, SP Sepharose FF, Cu-Ida Sepharose FF, Butyl Sepharose FF, Phenyl Sepharose FF, and Superdex 200. Application of CHT Ceramic Hydroxyapatite type II column combined with a gradient elution with sodium phosphate demonstrated the best separation of impurities. A strong cation exchanged chromatography on the SP Sepharose FF column was selected for the final purification step of the recombinant proteins. This step allows for obtaining the target protein into the sodium acetate buffer (pH 4.7) suitable for storage. A flowchart of the purification steps is presented in Fig. 2A. The summary of the yield and purity level of proteins after each purification step is presented in Table 1. The recovered total amount of SCF-Lα-GCSF and GCSF-Lα-SCF was 2.8 mg and 2.6 mg, that represents a yield of about 1.2% and 1.4%, respectively (Table 1).

**Table 1:** Yield and purity of SCF-Lα-GCSF and GCSF-Lα-SCF after four processing steps.

Protein purification step	Characteristic (%)	SCF-Lα-GCSF	GCSF-Lα-SCF
Refolding	Yield^1^	93.2 ± 2.9	91.7 ± 2.1
	Purity^2^	24.2 ± 1.0	35.8 ± 2.2
I Chromatography step (anion exchange)	Yield^1^	7.3 ± 0.5	6.1 ± 0.3
	Purity^2^	63.5 ± 2.5	58.6 ± 1.4
II Chromatography step (mixed-mode)	Yield^1^	1.9 ± 0.4	1.8 ± 0.3
	Purity^2^	77.5 ± 3.0	67.2 ± 2.2
III Chromatography step (cation exchange)	Yield^1^	1.4 ± 0.1	1.2 ± 0.2
	Purity^2^	92.2 ± 2.1	90.4 ± 1.6

**Notes:**

1 Protein concentrations were determined by the Bradford method (Bradford, 1976) using bovine serum albumin as a standard.

2 Purity of the protein was determined by RP-HPLC.

Each value represents the mean of three independent analyses ± standard deviation (SD).

### Characterization of the purified heterodimeric proteins

The purified proteins were characterized using a set of analytical methods including SDS-PAGE, Western-blotting, RP-HPLC, size exclusion HPLC (SE-HPLC) and HPLC/ESI-MS. The *E. coli* derived G-CSF and SCF monomers were used as the reference standards.

**Figure 3:**
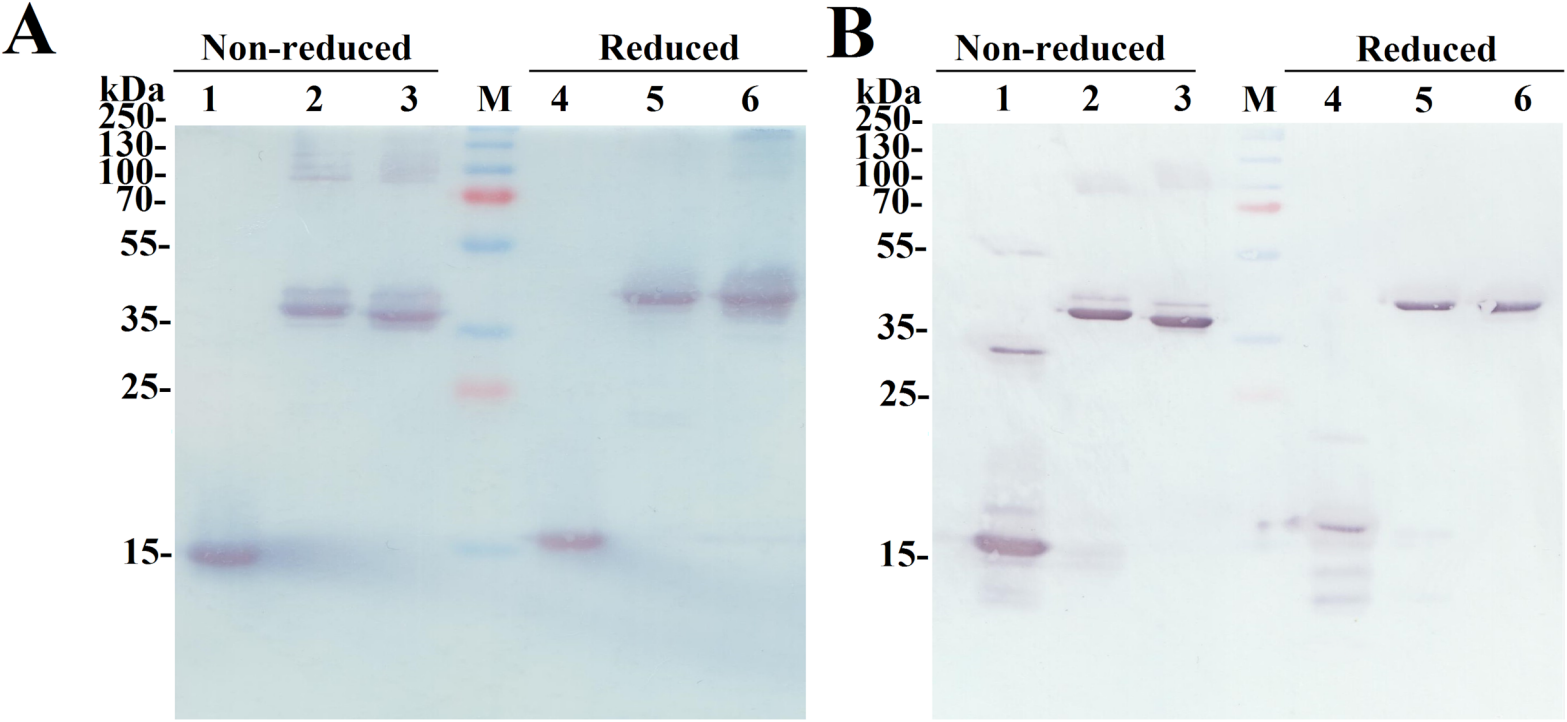
Identification of the purified fusion proteins with the antibodies in Western blot. The proteins were separated on a 15% SDS-PAGE under non-reducing and reducing conditions. (A) The Western blot with the monoclonal antibody against G-CSF. Lanes 1, 4, the G-CSF monomer; lanes 2, 5, SCF-Lα**-**GCSF; lanes 3, 6, GCSF-SCF. (B) The Western blot with polyclonal antibodies against SCF. Lanes 1, 4, the SCF monomer; lanes 2, 5, SCF-Lα-GCSF; lanes 3, 6, GCSF-Lα-SCF. Lane M, prestained molecular weight marker (Thermo Fisher Scientific, Waltham, MA, USA).

The SDS-PAGE analysis of the reduced and non-reduced heterodimers is shown in Fig. 2C. The reduced SCF-Lα-GCSF and GCSF-Lα-SCF proteins were detected as a single band on the gel. The respective bands were observed at the positions that corresponded to the molecular weight of the proteins (42 kDa). The non-reduced SCF-Lα-GCSF and GCSF-Lα-SCF produced the bands that were observed at lower positions (39 and 37 kDa, respectively) on the gel than their calculated molecular weight. Some heterogeneity in the non-reduced protein preparations indicated the presence of minor bands throughout the purification process (Fig. S2). Overall, a set of higher molecular weight products in protein preparations were detected both on the SDS-PAGE gel and by Western blotting with polyclonal anti-SCF and monoclonal anti-G-CSF antibodies (Fig. 3). The RP-HPLC profiles showed different hydrophobicity of SCF-Lα-GCSF and GCSF-Lα-SCF (Fig. 2B), whereas the degree of purity was nearly the same (92.2% and 90.4%, respectively) (Table 1).

**Figure 4:**
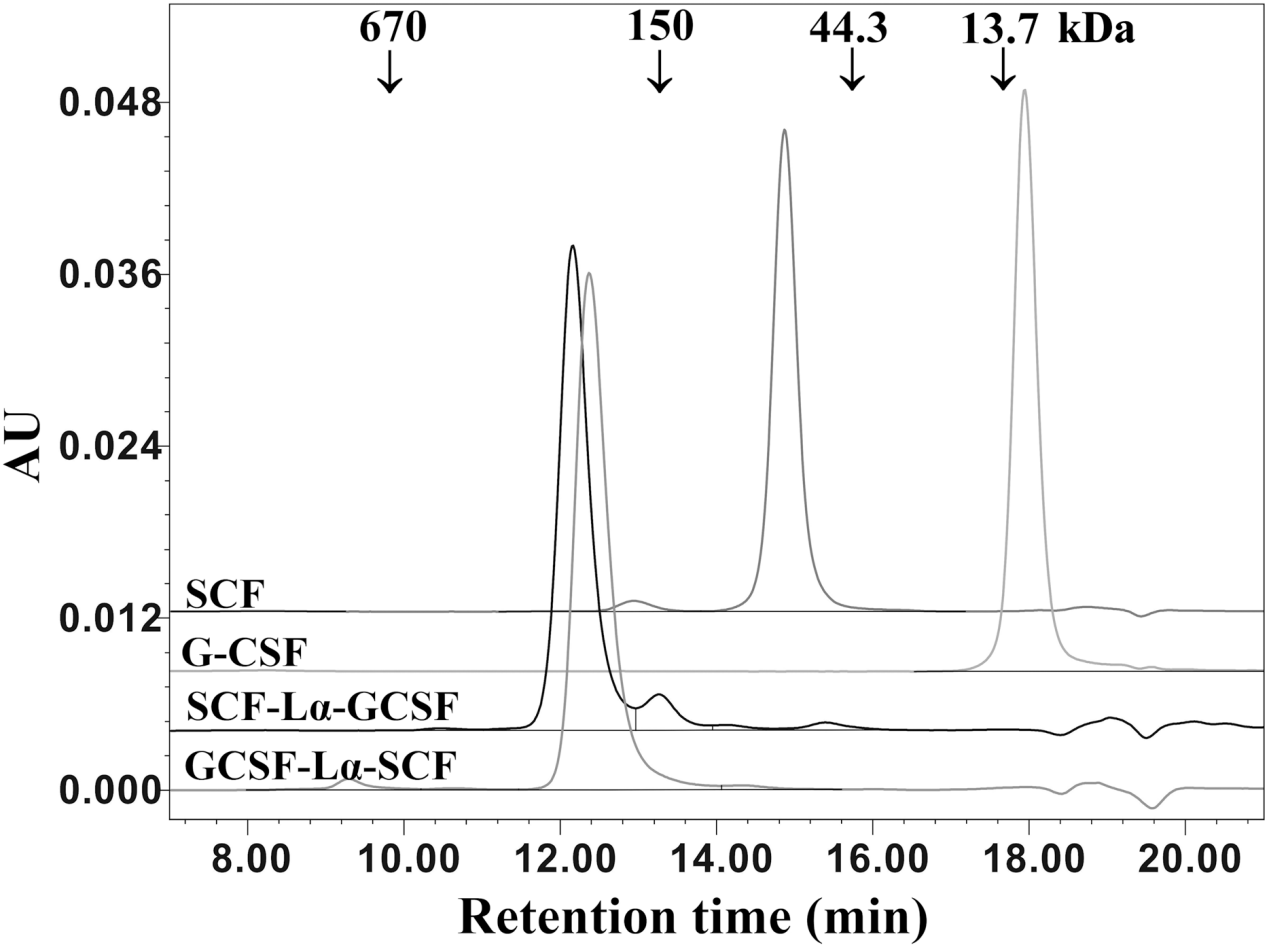
SE-HPLC analysis of purified SCF-Lα-GCSF and GCSF-Lα-SCF. Ten-µg of each protein was loaded onto a TSK-gel G3000 SWXL column. The column was calibrated with a protein standard mix (Sigma–Aldrich, St. Louis, MO, USA). The SCF and G-CSF monomers were used as controls. Absorbance at 280 nm is reported as AU.

The oligomeric state and molecular weight of the fusion proteins were analyzed by the calibrated SE-HPLC at pH 7.2 (Fig. 4). G-CSF was eluted as a monomer with a molecular weight lower than 13.7 kDa, whereas SCF was detected as a dimeric protein (>44.3 kDa). Both SCF-Lα-GCSF and GCSF-Lα-SCF were found as significantly higher molecular weight (>150 kDa) proteins than predicted (~42 kDa). The SE-HPLC data (Fig. 4) showed a tendency of heterodimeric proteins to form multimeric structures, presumably elongated non-covalently associated dimers or trimers without a monomeric fraction.

**Figure 5:**
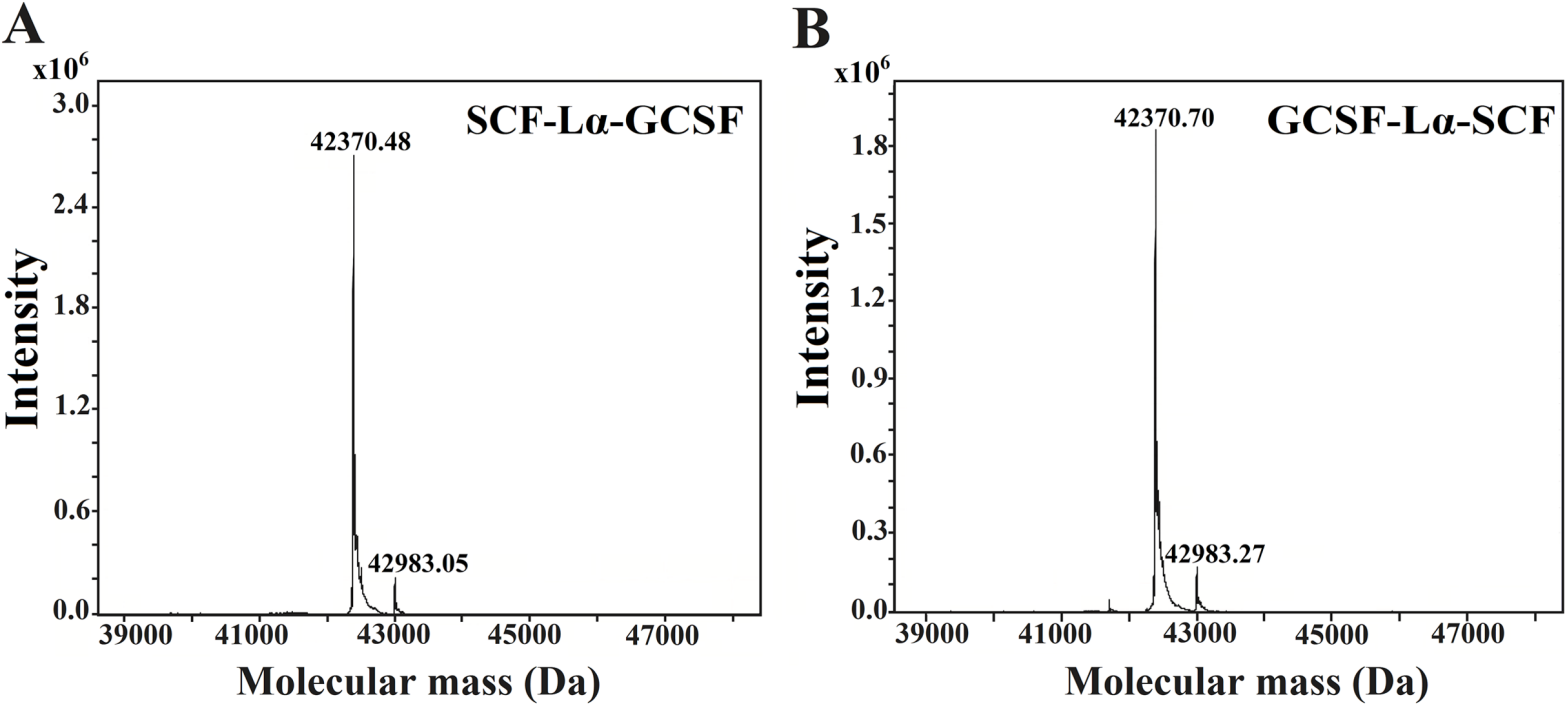
Molecular mass determination of the purified SCF-Lα-GCSF (A) and GCSF-Lα-SCF (B) proteins by mass spectrometry.

The HPLC/ESI-MS analysis revealed the composition and molecular mass of purified heterodimers (Fig. 5). Some heterogeneity in the monomeric preparations of SCF-Lα-CSF and GCSF-Lα-SCF was observed. The major peaks in the HPLC/ESI-MS chromatograms corresponded to 42,370.48 Da for SCF-Lα-GCSF (Fig. 5A) and 42,370.70 Da for GCSF-Lα-SCF (Fig. 5B), whereas the minor peaks corresponded to a mass of 42,983.05 and 42,983.27 Da, respectively. The detected molecular mass of both proteins was in good agreement with that of deduced from their amino acid sequences (within a range of 1 Da of the theoretical value).

### Binding kinetics of the heterodimeric proteins to the immobilized receptors

Binding of SCF-Lα-GCSF and GCSF-L-SCF to the SAM-immobilized receptors, GCSF-R or c-kit, was analyzed using TIRE. The obtained binding kinetics were compared to that of monomeric SCF and G-CSF. To improve the sensitivity of the ligand binding platform, the receptors were oriented perpendicular to the sensor’s surface. This “site-directed” immobilization was realized by the formation of the G protein-based SAM layer and the receptor layer (GCSF-R or c-kit) (Balevicius et al., 2014, 2019). To analyze the kinetics of the protein–protein interaction, a standard fully reversible Langmuir kinetic model was applied (Balevicius et al., 2014, 2019).

The kinetics of the SCF-Lα-CSF, GCSF-L-SCF, and SCF binding to the receptors are presented in Table 2 and Fig. S3. Binding kinetics of monomeric G-CSF and SCF-Lα-GCSF to GCSF-R were obtained from our previous study (Balevicius et al., 2019). The association rate constant *k*a between SCF-Lα-GCSF and c-kit was about 6-fold higher than that of the SCF monomer, whereas *k*a was 80-fold higher for GCSF-Lα-SCF. The dissociation rate constant *k*d for SCF-Lα-CSF was similar to that of the SCF monomer, although it was approx. 45-fold higher for GCSF-Lα-SCF. The *k*a and *k*d between SCF-Lα-GCSF and GCSF-R were 10-fold lower than that of monomeric G-CSF (Balevicius et al., 2019). Binding of GCSF-Lα-SCF to GCSF-R was not observed (Fig. S3D).

**Table 2:** Summary of characteristics of the fusion proteins and respective monomers.

Characteristic	SCF-Lα-GCSF	GCSF-Lα-SCF	G-CSF monomer	SCF monomer
Theoretical MW (kDa)	4,2369.74	4,2369.74	18,798.85	18,656.49
Experimental MW (kDa)	4,2370.48	4,2370.70	ND	ND
SEC size (kDa)	>150	>150	<13.7	>44.3
Biological activity on G-NFS-60 cell line^a^ (IU/mmol)	0.63 × 10^12^	0.25 × 10^12^	1.88 × 10^12^	ND
Biological activity on M-07e cell line^b^ (IU/mmol)	6.70 × 10^9^	12.80 × 10^9^	ND	9.33 × 10^9^
Association and dissociation constants of the protein and the SCF receptor (TIRE data)				
*k*a (M^−1^ s^−1^)	7.77 × 10^4^	1.14 × 10^6^	ND	1.37 × 10^4^
*k*d (s^−1^)	1.39 ×10^−2^	6.46 × 10^−1^	ND	1.43 × 10^−2^
K_a_ (M^−1^)	5.59 × 10^6^	1.76 × 10^6^	ND	9.50 × 10^5^
(K_d_) (M)	1.78 × 10^−7^	5.67 × 10^−7^	ND	1.04 × 10^−6^
Association and dissociation constants of the protein and the G-CSF receptor (TIRE data)				
*k*a (M^−1^ s^−1^)	8.50 × 10^4^^c^	No interaction	7.50 × 10^5^^c^	ND
*k*d (s^−1^)	1.25 × 10^−3^^c^		1.05 × 10^−2^^c^	ND
K_a_ (M^−1^)	6.80 × 10^7^^c^		7.14 × 10^7^^c^	ND
K_d_ (M)	0.15 × 10^−7^^c^		0.14 × 10^−7^^c^	ND

**Notes:**

a Cell line expressing the G-CSF receptor.

b Cell line expressing the SCF receptor.

c The data previously described in Balevicius et al. (2019).

ND, not determined.

### Biological activity in vitro and in vivo

**Figure 6:**
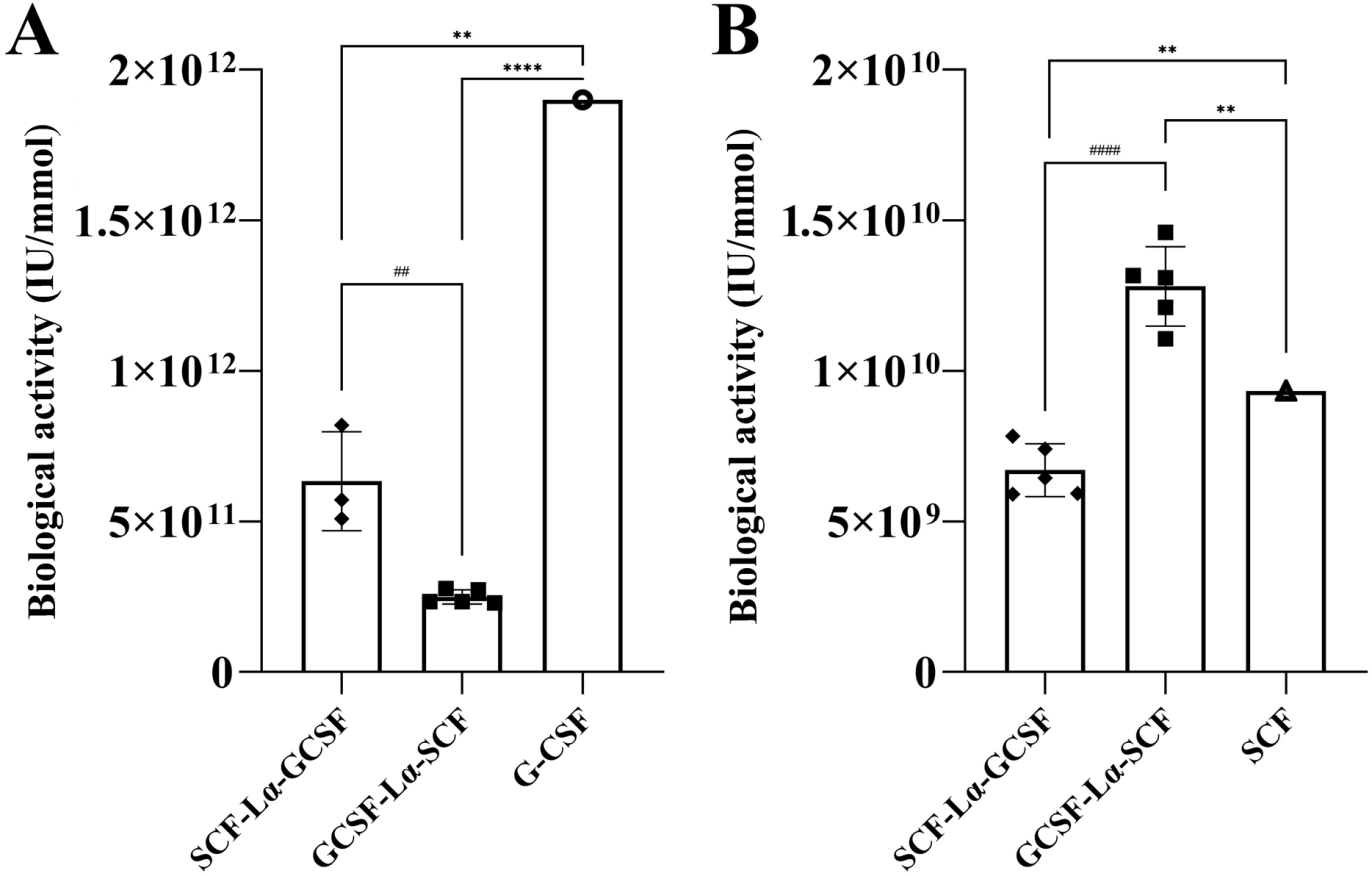
The proliferation of G-NFS-60 (A) and M-07e (B) cells induced by the purified SCF-Lα-GCSF and GCSF-Lα-SCF. The plots represent the specific biological activity of the heterodimers calculated from proliferation curves using the equations as described previously (Mickiene et al., 2017). Results are expressed as the mean ± standard deviation (SD). Individual values from the independent assays are indicated by the squares. * Values are significantly different between the biological activity of heterodimer and monomer (the one sample *t*-test, **p ≤ 0.01 and ****p ≤ 0.0001). Biological activity of G-CSF and SCF are provided by the manufacturers (indicated by the circle and triangle). #Values are significantly different between the biological activity of SCF-Lα-GCSF and GCSF-Lα-SCF (the unpaired *t*-test, ##*p* ≤ 0.01 and ####*p* ≤ 0.0001).

The in vitro biological activity of the SCF and G-CSF moieties in each fusion protein were evaluated by the proliferation assays of M-07e cells expressing the SCF receptor, but not the G-CSF receptor and the G-NFS-60 cells expressing the G-CSF receptor, but not the SCF receptor. The fusion proteins induced a dose-dependent proliferative response on both cell lines (Figs. S4 and S5). The calculated activity of SCF-Lα-GCSF on the M-07e cells reached 72% (*p* ≤ 0.01), whereas GCSF-Lα-SCF showed a relative activity of 137% (*p* ≤ 0.01) of that of monomeric SCF at the equimolar amount (Fig. 6B; Table 2). Differences in the proliferative response on M-07e cells between the heterodimers reached statistical significance (*p* ≤ 0.0001). The proliferative response of SCF-Lα-GCSF and GCSF-Lα-SCF on the G-NFS-60 cells reached 34% (*p* ≤ 0.01) and 13% (*p* ≤ 0.0001), respectively, compared to that of the monomeric form of G-CSF at the equimolar amount (Fig. 6A; Table 2). Notably, no binding of GCSF-Lα-SCF to the immobilized GCSF-R was detected by the TIRE method.

**Figure 7:**
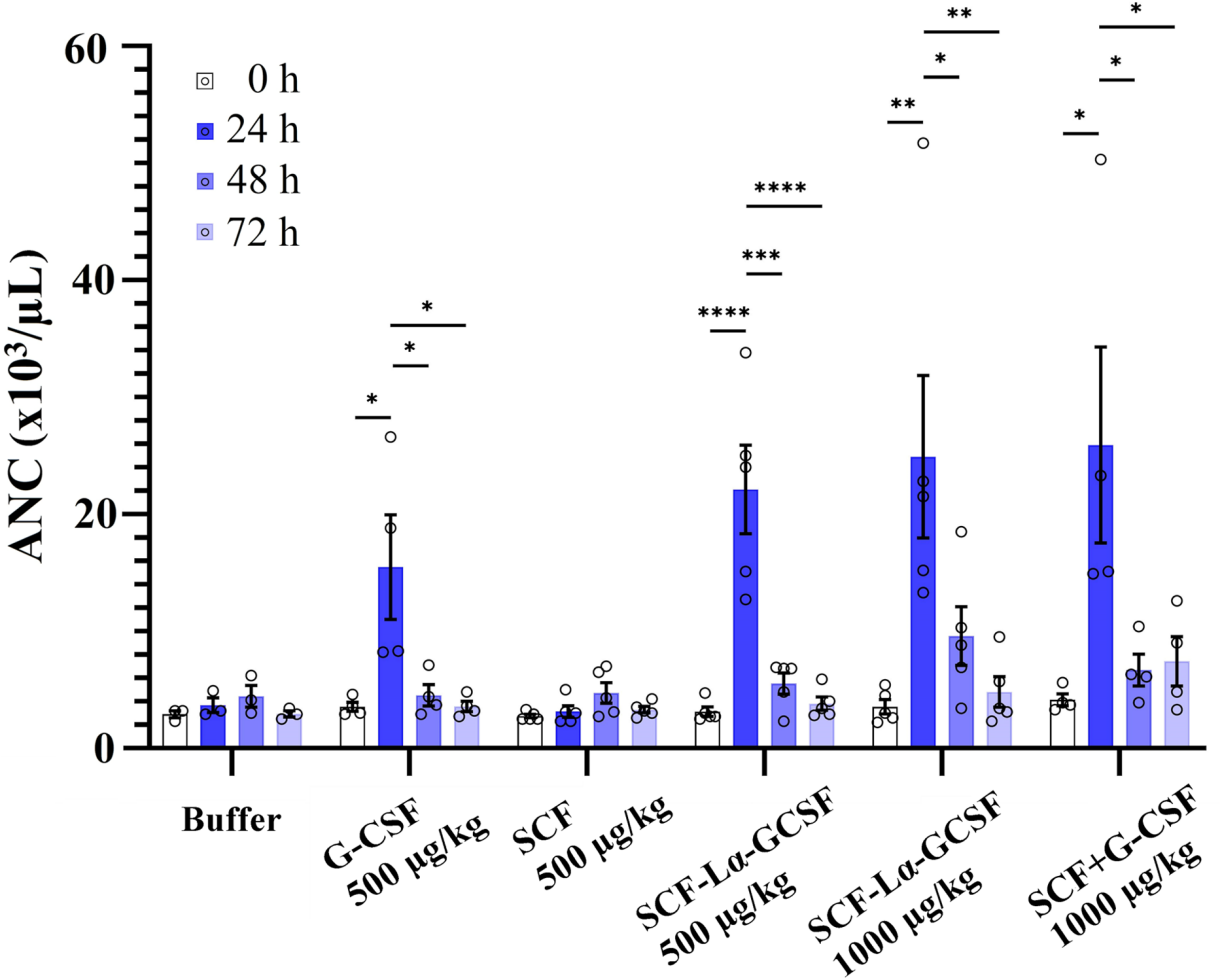
The ANC count versus time profiles obtained by subcutaneously administered recombinant proteins. Each rat in the respective group received injections of SCF-Lα-GCSF (500 or 1,000 µg/kg), G-CSF (500 µg/kg), SCF (500 µg/kg), a mixture of SCF and G-CSF (500 + 500 µg/kg), and a control buffer. Results are expressed as the mean ± SEM. Individual values from the independent assays are indicated by the open circles. A one-way ANOVA combined with the Tukey’s multiple comparison test was performed to compare the means of ANC values at different time points in each group (buffer or protein). *Values are significantly different between ANC count induced by injection of each protein at different time points (*p < 0.05; **p < 0.01; ***p < 0.001, ****p < 0.0001).

The GCSF-Lα-SCF protein showed a significantly lower activity of the G-CSF moiety in vitro than that of SCF-Lα-GCSF (*p* ≤ 0.01), therefore the latter was used for in vivo studies. G-CSF stimulates neutrophil release from the bone marrow inducing a transient increase in circulated neutrophils. The biological activity of the G-CSF moiety in vivo was tested by the detection of ANC in the peripheral blood of rats in six groups. The ANC peaked 24 h after the subcutaneous administration of SCF-Lα-GCSF (1,000 µg/kg) demonstrating a 7-fold increase (*p* ≤ 0.01) compared to ANC before the injection, whereas a mixture of G-CSF and SCF (500 + 500 µg/kg) showed a 6.3-fold increase (*p* ≤ 0.05) (Fig. 7). The monomeric G-CSF at equimolar concentration showed a 4.4-fold higher ANC count (*p* ≤ 0.05), whereas no statistically significant difference was detected after the injection of SCF compared to ANC before the injection. There is a visible trend that a drop of ANC did not occur immediately at 48 and 72 h post-injection of the heterodimer (a 3-fold (*p* < 0.05) and 5-fold (*p* < 0.01) decrease, respectively compared to ANC at 24 h post-injection) or a mixture of G-CSF and SCF (a 4-fold (*p* < 0.05) and 3-fold (*p* < 0.05) decrease, respectively compared to ANC at 24 h post-injection) (Fig. 7). It was observed a 4-fold decrease in ANC (*p* < 0.05) at 48 h post-injection of G-CSF compared to ANC at 24 h post-injection (Fig. 7).

## Discussion

The short half-life of biopharmaceuticals is conditioned by a rapid metabolism, proteolytic degradation, and susceptibility of small proteins to renal clearance (Kontermann, 2011). Chemical modification and covalent attachment of polyethylene glycol (PEG) are among half-life extension strategies dedicated to increasing the hydrodynamic volume of the protein (Kontermann, 2011; Van Witteloostuijn, Pedersen & Jensen, 2016). PEGylation has found a wide application for extending the circulating half-life of many protein drugs, however, the safety of PEGylated compounds and PEG itself is still under consideration (Zhang, Liu & Wan, 2014). The concerns are connected with the antibody formation against the PEG moiety, the greater potential of PEGylated proteins for accumulation in the cells, and heterogeneity of PEGylated proteins (Zhang, Liu & Wan, 2014; Baumann et al., 2014). The fusion of therapeutic proteins to Fc and human serum albumin results in a prolonged half-life and allows overcoming problems connected with PEGylation (Strohl, 2014). Overall, the multimeric proteins produced by more simple technology than PEGylation have become attractive therapeutics due to increased biological activity and/or prolonged circulation time (Czajkowsky et al., 2012; Strohl, 2014).

In this study, we presented generation, purification and characterization of two fusion heterodimeric proteins SCF-Lα-GCSF and GCSF-Lα-SCF. The Lα linker used to fuse SCF and G-CSF was selected, based on the previous research (Mickiene et al., 2017). It was shown that the α-helix conformation of the linker efficiently separates the domains of the bifunctional fusion proteins ensuring that the distance between the monomers is favorable for their independent functioning (Arai et al., 2001; Chen, Zaro & Shen, 2013).

Two fusion proteins were expressed in *E. coli*, recovered from the inclusion bodies with subsequent refolding by dilution, and purified using an anion-exchange, mixed-mode, and cation-exchange chromatography (Fig. 2A).

A purification yield reaching more than 2.6 mg/12 g wet cell mass was obtained for both fusion proteins. The purity of proteins was more than 90% determined by RP-HPLC. The results of SDS-PAGE and Western blot showed that the heterodimer preparations represented a homogeneous protein under reducing conditions. Under non-reducing conditions, the amount of monomeric forms of SCF-Lα-CSF and GCSF-Lα-SCF (39 and 37 kDa, respectively) decreased and the higher molecular weight protein bands on the gel were detected. These extra bands found throughout the purification process showed reactions with the monoclonal antibody against G-CSF and the polyclonal antibodies against SCF. It was reported that *E. coli*-derived human SCF produced a major SDS-dissociable form, that is, similar to naturally occurring SCF, whereas a minor form is a non-SDS-dissociable disulfide-linked dimer (Zsebo et al., 1990; Lu et al., 1996; Hsu et al., 1997*)*. The presence of the disulfide-linked multimeric forms (range of 87–120 kDa) in the preparations of heterodimeric proteins corresponded to that of *E. coli*-derived SCF.

The ESI/MS analysis confirmed the calculated molecular mass of the fusion proteins, although additional minor peaks corresponding to the molecular mass of 42,983.05 Da for SCF-Lα-GCSF and 42,983.27 Da for GCSF-Lα-SCF were detected. The molecular mass difference between the minor and major peaks suggests that the impurities may represent a heterodimer with an open disulfide bond (Taniuchi et al., 1977) that results in the association of cysteines with two reduced molecules of glutathione.

This assumption might explain the occurrence of the extra minor band with a molecular weight of 47 kDa for SCF-Lα-GCSF and 45 kDa for GCSF-Lα-SCF on SDS-PAGE under non-reducing conditions. The SE-HPLC analysis showed that both fusion proteins have comparable tertiary structures and the estimated molecular mass was higher than expected. The SCF preparations showed a similar abnormal elution profile (Arakawa et al., 1991). At neutral pH, the apparent molecular weight (57 kDa) of *E. coli-*derived SCFs was related to a relatively large Stoke radius. The lower molecular mass fraction of the heterodimers on the SDS-PAGE gel indicates that the fusion proteins are non-covalent dimers or trimers under native conditions.

The biochemical activity of SCF and G-CSF moieties in each fusion protein was evaluated analyzing the interaction between the heterodimers and their SAM-immobilized receptors. GCSF-Lα-SCF exhibited more efficient binding to the SCF receptor than the SCF-Lα-GCSF protein (Table 2). Thus, the G-CSF moiety on either GCSF-Lα-SCF or SCF-Lα-GCSF did not affect the binding of the SCF moiety to its receptor. The N-terminal SCF moiety of SCF-Lα-GCSF did not impair the C-terminal G-CSF moiety’s binding with R-GCSF. However, the C-terminal SCF moiety of GCSF-Lα-SCF blocked the N-terminal G-CSF binding to its receptor. Each fusion protein promoted the M-07e cell proliferation. The potency of GCSF-Lα-SCF was about 1.4-fold higher than that of monomeric SCF at equimolar concentration, whereas SCF-Lα-GCSF induced cell proliferation at a significantly lower level. The fusion proteins showed a remarkably reduced proliferative response of the G-CSF moiety on the G-NFS-60 cell line. The structural studies showed that the N-terminal residues and the Cys4-Cys89 disulfide bond of SCF are required for a receptor binding and activity (Zhang et al., 2000; Jiang et al., 2000). However, the N-terminal G-CSF moiety of GCSF-Lα-SCF did not reduce binding of SCF to its receptor. The results indicate that the Lα linker ensured spatial separation between the domains of the fusion protein at a favorable distance for their independent functioning. The reduced G-CSF activity of two fusion proteins may be due to a steric hindrance caused by the multimeric forms of the SCF moiety.

In vivo, SCF-Lα-GCSF stimulated a 7.0-fold increase of ANC and the effect was similar to that of the mixture of SCF and G-CSF (a 6.3-fold increase), whereas the response induced by the G-CSF monomer was a 4.4-fold higher at equimolar concentration. Human SCF has low bioactivity on rodent hematopoietic cells (Fitzgerald et al., 2001; Broudy, 1997), therefore the obtained data does not properly reflect the potency of the SCF-Lα-GCSF heterodimer in vivo. The promising in vitro and in vivo activity of SCF-Lα-G CSF motivates looking for the models suitable for demonstration of the synergistic effect of G-CSF and SCF moieties in the fusion protein.

## Conclusions

In this study, two heterodimeric proteins were generated by a covalent fusion of human SCF and human G-CSF proteins via the flexible Lα linker. The SCF monomer in both proteins gained a proper conformation, while the linker ensured distance favorable for its independent functioning. However, an array of the SCF and G-CSF moieties in the fusion proteins had an impact on the respective ligand–receptor interaction that resulted in a different activity of proteins in promoting cell proliferation. The SCF-Lα-GCSF protein is a promising candidate for further studies due to the biological activity in vivo comparable to that of the mixture of SCF and G-CSF.

## Supplemental Information

10.7717/peerj.9788/supp-1 Supplemental Information 1 The RP-HPLC analysis of SCF-GCSF and GCSF-SCF preparations. (A) The protein extracts from inclusion bodies in the absence or presence of 0.5 mM DTT. (B) Protein samples after refolding in the presence of the DTT/GSSG couple. Each protein sample was loaded onto a Zorbax 300SB-C18 column (Agilent Technologies, Santa Clara, CA, USA). Absorbance at 215 nm is reported as AU. *Represents the oxidized protein form.

10.7717/peerj.9788/supp-2 Supplemental Information 2 The SDS-PAGE analysis of SCF-Lα-GCSF (A and B) and GCSF-Lα-SCF (C and D) preparations obtained through different purification steps under non-reducing and reducing conditions. Lane 1, the protein extract from inclusion bodies, in the presence of urea and DTT; Lane 2, protein sample after refolding in the presence of DTT/GSSG couple; Lane 3, the pool of fractions recovered from the DEAE Sepharose FF column; Lane 4, the pool of fractions recovered from the CHT ceramic hydroxyapatite type II column; Lane 5, the final product obtained after application of a SP Sepharose FF chromatography. Lane M, ****prestained molecular weight marker (Thermo Fisher Scientific).

10.7717/peerj.9788/supp-3 Supplemental Information 3 Interaction between the monomeric/heterodimeric proteins and the oriented SAM-immobilized receptors. (A) monomeric SCF-c and c-kit. (B) SCF-Lα-GCSF and c-kit. (C) GCSF-Lα-SCF and c-kit. (D) GCSF-Lα-SCF and GCSF-R. The protein–receptor interaction was started to record immediately after protein injection (label by an arrow) into the TIRE cell. After 20 min the cell was flushed (labeled by an arrow) with the protein-free PBS buffer to remove unbound protein. F, normalized analytical signal.

10.7717/peerj.9788/supp-4 Supplemental Information 4 Effects of purified SCF-Lα-GCSF (A) and GCSF-Lα-SCF (B) on the proliferation of G-NFS-60 cells. The G-CSF monomer (control) included in each assay. The curves were obtained at two-fold doubling (log 2) serial dilutions of the tested proteins. Error bars represent standard deviation (SD) of the absorbance means (490 nm) obtained in 3–5 independents assays.

10.7717/peerj.9788/supp-5 Supplemental Information 5 Effects of purified SCF-Lα-GCSF (A) and GCSF-Lα-SCF (B) on the proliferation of M-07e cells. The SCF monomer (control) included in each assay. The curves were obtained at two-fold doubling (log 2) serial dilutions of the tested proteins. Error bars represent standard deviation (SD) of the absorbance means (490 nm) obtained in five independent assays.

10.7717/peerj.9788/supp-6 Supplemental Information 6 Raw data for Figs. 1 and 2.

10.7717/peerj.9788/supp-7 Supplemental Information 7 Raw data for Table 1.

10.7717/peerj.9788/supp-8 Supplemental Information 8 Raw data of Figure 5.

10.7717/peerj.9788/supp-9 Supplemental Information 9 Raw data for Fig. 6.

10.7717/peerj.9788/supp-10 Supplemental Information 10 Raw data for Fig. 7.

10.7717/peerj.9788/supp-11 Supplemental Information 11 Raw data for Fig. S2.

10.7717/peerj.9788/supp-12 Supplemental Information 12 Raw data for Fig. S3.

10.7717/peerj.9788/supp-13 Supplemental Information 13 Raw data for Figs. S4 and S5.

## Additional Information and Declarations

### Field Study Permissions

The following information was supplied relating to field study approvals (i.e., approving body and any reference numbers): State Food and Veterinary Service of the Republic of Lithuania (approval no. 0182).

### Data Availability

The following information was supplied regarding data availability: The raw data are available as Supplemental Files.
