# Supplementary figures and images for: Human granulocyte-colony stimulating factor (G-CSF)/stem cell factor (SCF) fusion proteins: design, characterization and activity
Source: PeerJ. 2020 Aug 21;8:e9788. doi: 10.7717/peerj.9788 (PMC7444511; doi:10.7717/peerj.9788)

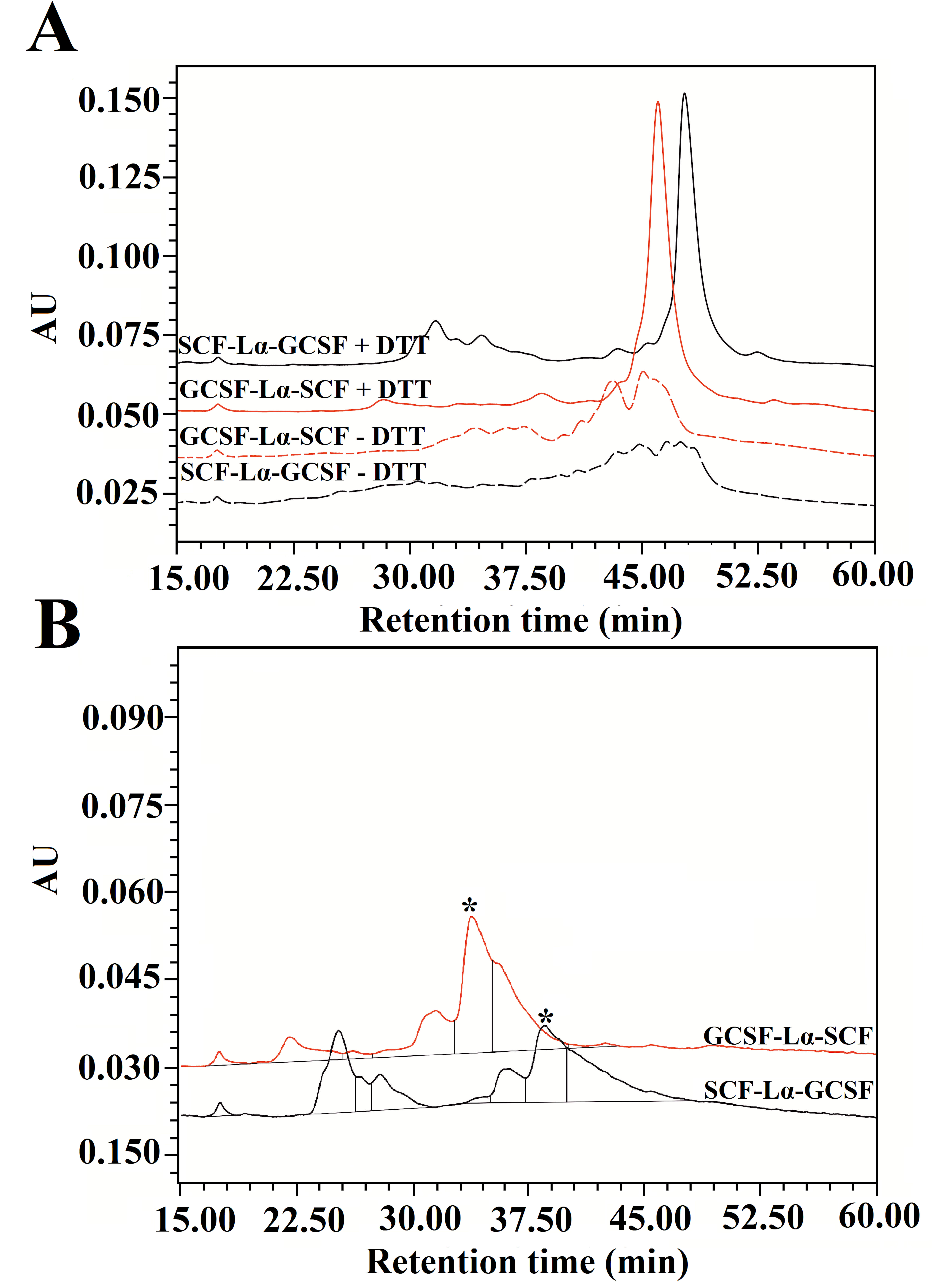

Supplement: Supplemental Information 1 — (A) The protein extracts from inclusion bodies in the absence or presence of 0.5 mM DTT. (B) Protein samples after refolding in the presence of the DTT/GSSG couple. Each protein sample was loaded onto a Zorbax 300SB-C18 column (Agilent Technologies, Santa Clara, CA, USA). Absorbance at 215 nm is reported as AU. *Represents the oxidized protein form. [file peerj-08-9788-s001.jpg]

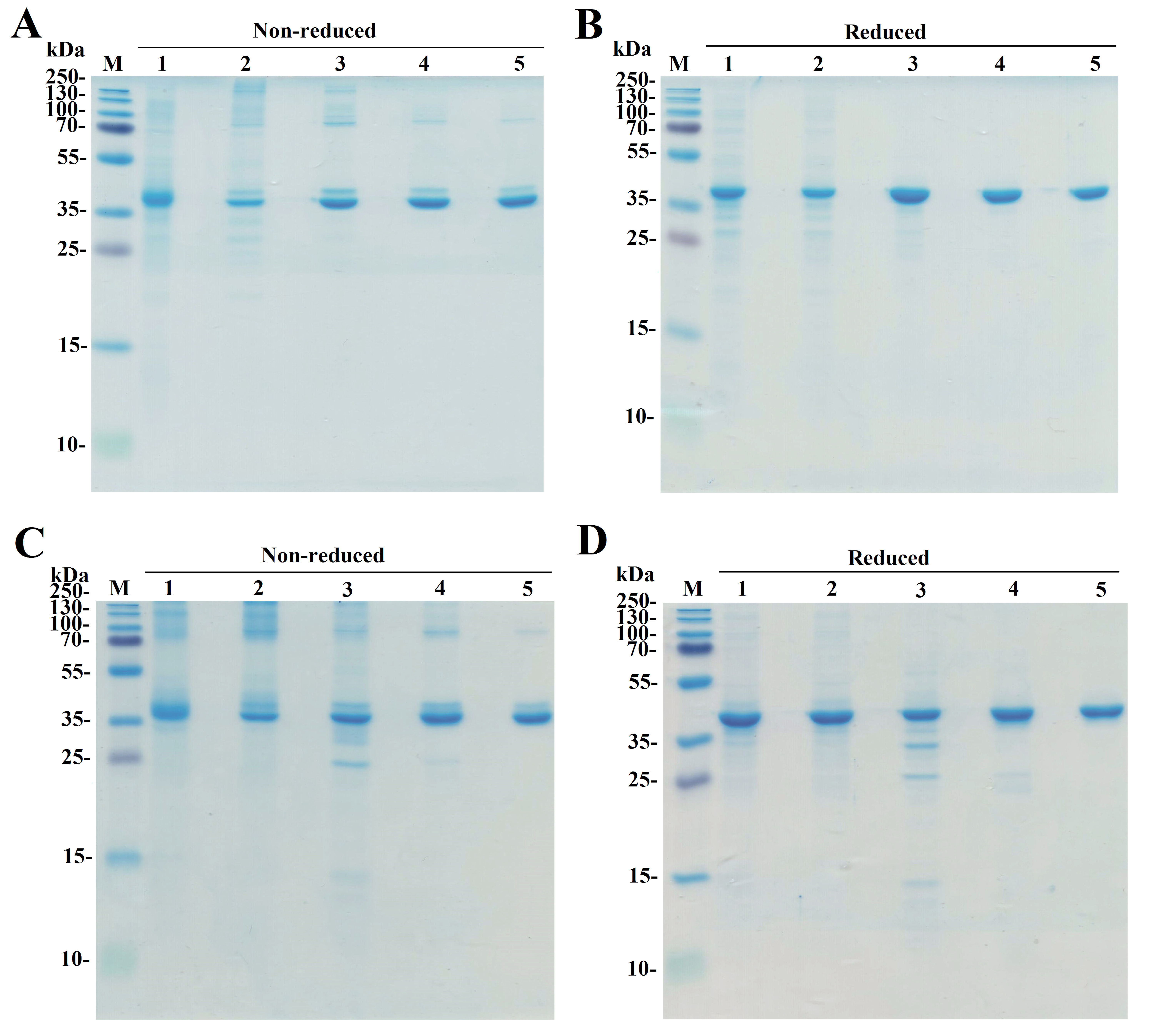

Supplement: Supplemental Information 2 — Lane 1, the protein extract from inclusion bodies, in the presence of urea and DTT; Lane 2, protein sample after refolding in the presence of DTT/GSSG couple; Lane 3, the pool of fractions recovered from the DEAE Sepharose FF column; Lane 4, the pool of fractions recovered from the CHT ceramic hydroxyapatite type II column; Lane 5, the final product obtained after application of a SP Sepharose FF chromatography. Lane M, prestained molecular weight marker (Thermo Fisher Scientific). [file peerj-08-9788-s002.jpg]

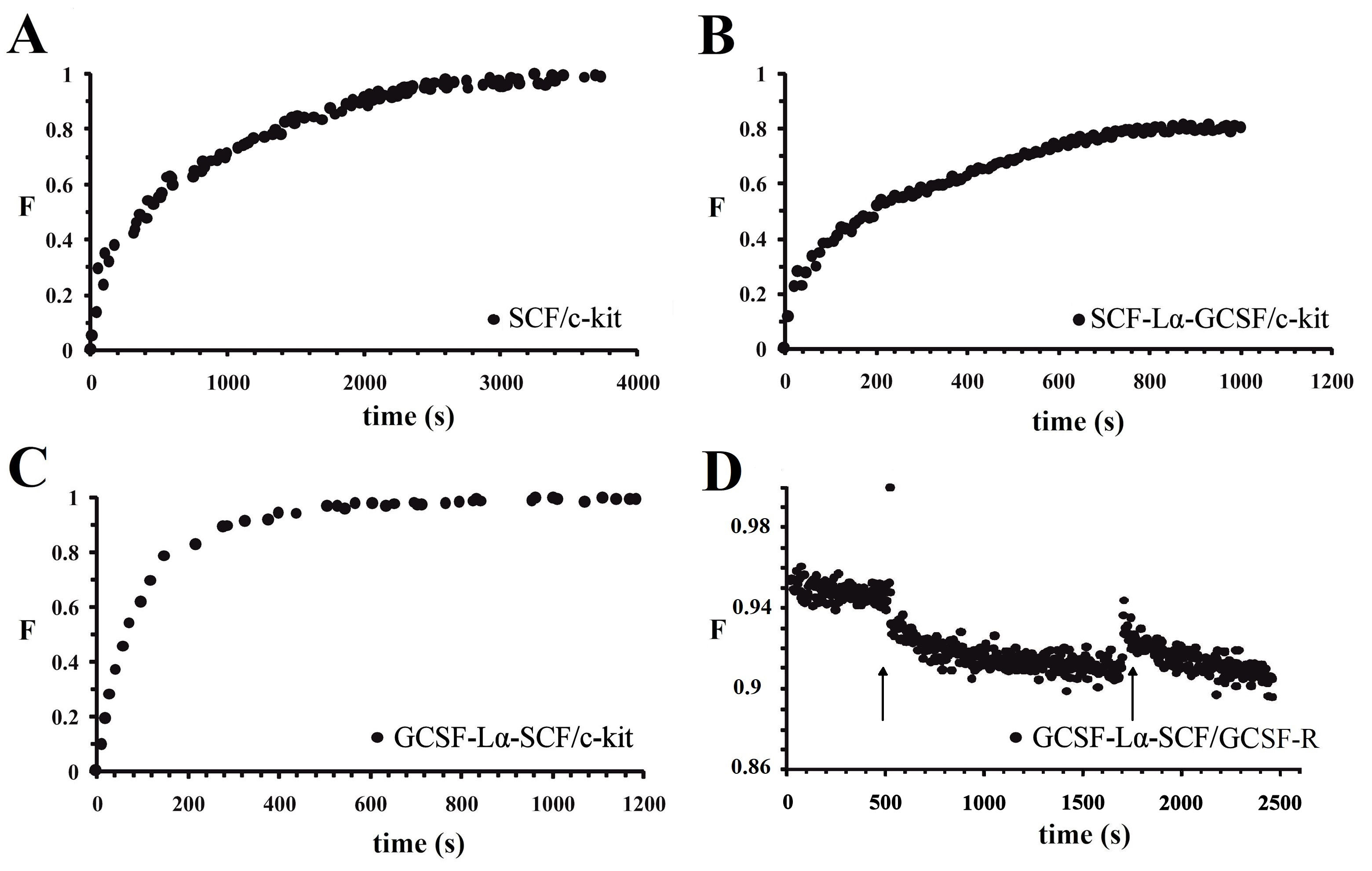

Supplement: Supplemental Information 3 — (A) monomeric SCF-c and c-kit. (B) SCF-Lα-GCSF and c-kit. (C) GCSF-Lα-SCF and c-kit. (D) GCSF-Lα-SCF and GCSF-R. The protein–receptor interaction was started to record immediately after protein injection (label by an arrow) into the TIRE cell. After 20 min the cell was flushed (labeled by an arrow) with the protein-free PBS buffer to remove unbound protein. F, normalized analytical signal. [file peerj-08-9788-s003.jpg]

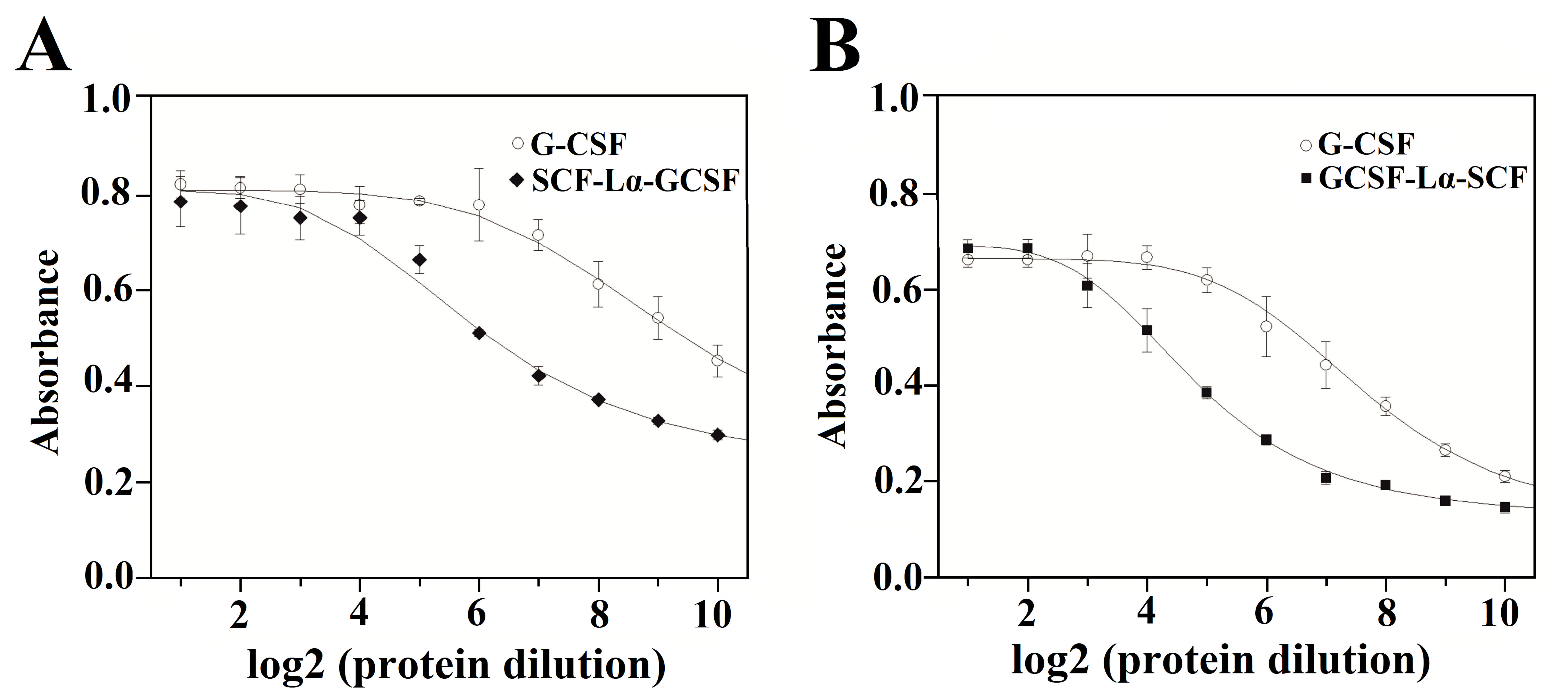

Supplement: Supplemental Information 4 — The G-CSF monomer (control) included in each assay. The curves were obtained at two-fold doubling (log 2) serial dilutions of the tested proteins. Error bars represent standard deviation (SD) of the absorbance means (490 nm) obtained in 3–5 independents assays. [file peerj-08-9788-s004.jpeg]

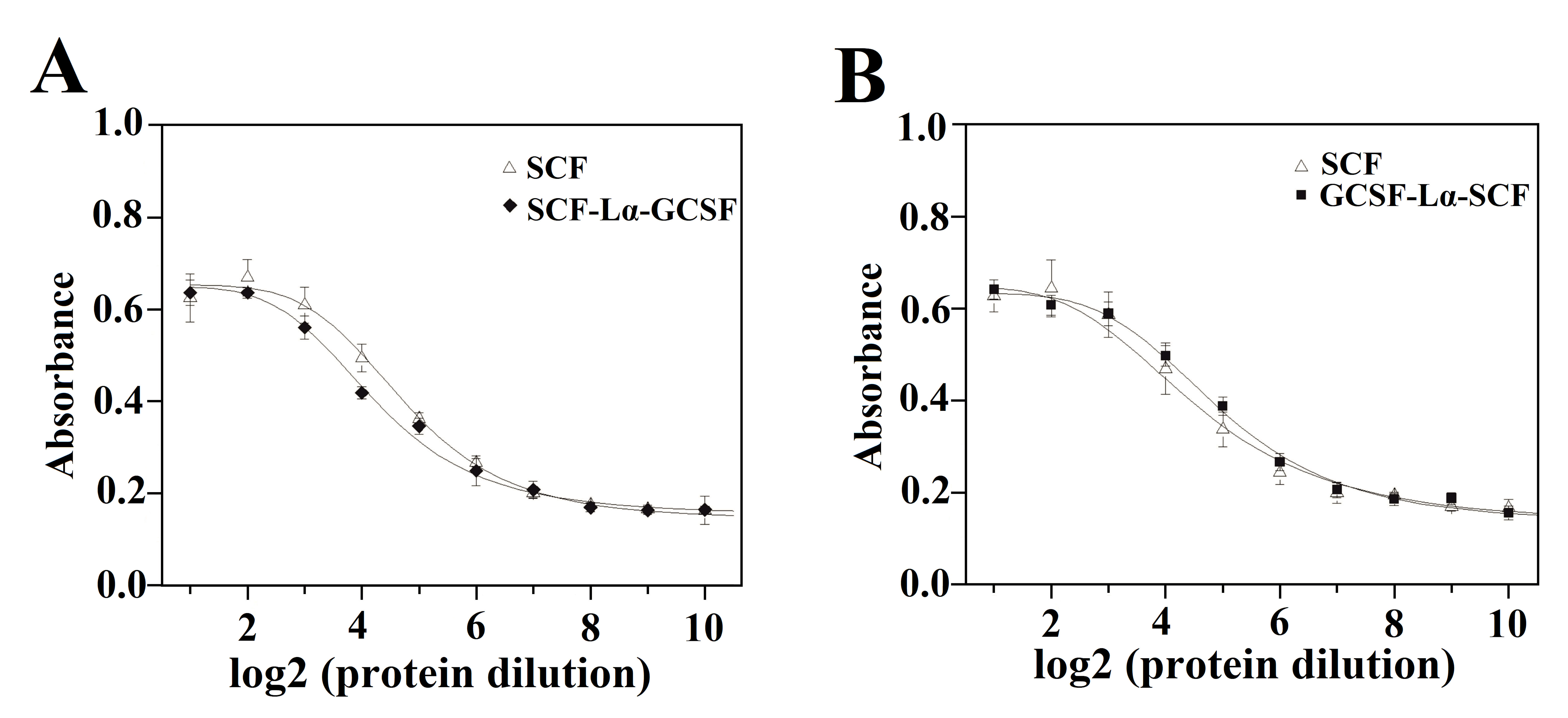

Supplement: Supplemental Information 5 — The SCF monomer (control) included in each assay. The curves were obtained at two-fold doubling (log 2) serial dilutions of the tested proteins. Error bars represent standard deviation (SD) of the absorbance means (490 nm) obtained in five independent assays. [file peerj-08-9788-s005.jpg]
